# Dynamic Analytical and Experimental Study of Wearable Thermoelectric Devices for Thermal Tactile Feedback

**DOI:** 10.3390/mi17060694

**Published:** 2026-06-05

**Authors:** Zhijia Cai, Aibing Zhang

**Affiliations:** Zhejiang-Italy Joint Lab for Smart Materials and Advanced Structures, School of Mechanical Engineering and Mechanics, Ningbo University, Ningbo 315211, China; 18757419863@163.com

**Keywords:** dynamic thermal conduction, analytical solutions, wearable thermoelectric devices, skin–WTED thermal interaction, thermal tactile feedback

## Abstract

Thermal tactile perception plays a crucial role in enhancing realism and immersion in human–machine interaction, virtual/augmented reality, and wearable systems. By exploiting the thermoelectric effect to achieve precisely controllable heating and cooling, wearable thermoelectric devices (WTEDs) offer an effective approach for generating localized and programmable thermal sensations, which calls for a clear understanding of skin temperature regulation mechanisms. In this work, a dynamic thermal conduction model is developed for a skin–WTED integrated system incorporating a nickel foam-reinforced hydrogel heat sink, based on the dual-phase lag (DPL) bioheat conduction theory. The model accounts for blood perfusion and metabolic heat generation in skin tissue, as well as the Thomson effect within the thermoelectric legs and convective heat losses from their side surfaces. The theoretical predictions are validated through human skin temperature regulation experiments using a fabricated WTED, showing close agreement between experiments and simulations and confirming the model’s accuracy and reliability. Based on the validated model, the cooling current, filling factor, and thermoelectric leg height are optimized by minimizing the skin surface temperature. Furthermore, the model is applied to thermal tactile feedback studies, enabling the controlled reproduction of skin thermal sensations associated with common objects, including an iron block, a PMMA plate, and carbonated beverages packaged in aluminum cans and plastic bottles. Overall, this study provides a practical and predictive framework for understanding, optimizing, and applying WTEDs in thermal tactile feedback.

## 1. Introduction

In virtual reality (VR) and augmented reality (AR) technologies, tactile interaction has become an increasingly important research topic [1,2,3]. Recent advances in flexible and skin-interfaced wearable devices, such as electronic skins and multifunctional tactile sensors, have significantly promoted the development of human–machine interfaces and wearable interaction systems [4]. Among various tactile modalities, thermal tactile perception plays a crucial role in environmental exploration and object recognition, enabling humans to avoid harmful extreme temperatures and to perceive changes in their surroundings [5,6,7,8]. The hand is one of the most sensitive regions of the human body because its skin contains diverse types of highly sensitive sensory receptors [9,10,11,12]. Notably, objects made of different materials can evoke distinct thermal sensations even when they are at the same temperature; for example, metal typically feels colder than wood. This phenomenon arises from the high sensitivity of thermoreceptors and the ability of the human nervous system to finely encode and process thermal stimuli, resulting in precise temperature perception [13,14,15]. These physiological characteristics provide a fundamental basis for the construction of artificial thermal tactile signals. By delivering controlled thermal stimuli during hand–object interactions, artificial thermal feedback can not only enhance user experience but also facilitate material identification through analysis of transient heat transfer processes at the skin–object interface [16], information that is often inaccessible through visual or auditory cues alone. In recent years, various wearable devices with controllable temperature outputs have been developed to transmit thermal information from virtual or augmented environments to users in the real world, thereby enabling realistic thermal tactile experiences [17]. Among existing approaches, Joule-heating-based devices have been widely adopted [18,19,20,21,22]; however, thermoelectric-based devices offer distinct advantages in temperature regulation accuracy, response speed, and bidirectional heating–cooling capability [23,24].

Thermoelectric devices (TEDs) enable energy conversion and thermal regulation based on thermoelectric effects and offer advantages such as compact size, light weight, absence of mechanical moving parts, and ease of control. As a result, they have been widely applied in thermal management and waste heat energy harvesting [25,26,27]. According to the Peltier effect, when an electric current passes through a TED, a temperature difference is generated across the device, and the direction of heat flow can be rapidly and reversibly switched by reversing the current direction [28,29]. Owing to this unique bidirectional temperature control and fast dynamic response, TEDs exhibit significant advantages in wearable thermal feedback and artificial thermal tactile rendering. Consequently, theoretical modeling and performance optimization studies of wearable thermoelectric devices (WTEDs) have increased in recent years [30,31,32,33,34]. Lee et al. [35] developed a WTED model to investigate the matching relationship between contact resistance and the optimal fill factor, showing that the influence of intrinsic contact resistance becomes more pronounced at lower fill factors. Wijethunge et al. [36] proposed a simplified human body temperature model for wearable thermoelectric applications, demonstrating variations in skin thermal resistance under external conditions. Treating skin tissue and WTEDs as a coupled integrated system, Zhang et al. [37,38] established a unified skin–WTED thermal analytical model to examine the effects of physiological factors, including blood perfusion, metabolic heat generation, and multilayer skin structure, on WTED power generation performance. Their results indicated that blood perfusion has a more significant impact on WTED performance than metabolic heat generation. Enescu [39] analyzed heat transfer mechanisms in WTEDs by characterizing the coupled thermal interactions between WTEDs and human skin, and classified wearable solutions based on active and passive thermal management strategies, natural and assisted heat exchange, autonomous and non-autonomous devices, as well as direct or indirect contact with the human body. Lee et al. [40] designed a WTED using a simplified human thermoregulation model to predict device performance. The theoretical results showed that, as a portable cooling device, the WTED could reduce the skin surface temperature by 5.4 K, while achieving a power density of 8.32 μW/cm^2^ for human body heat energy harvesting. Gabardi et al. [41] estimated transient heat exchange during skin–object contact using heat flux equations and, based on this analysis, developed a fingertip tactile device capable of delivering full tactile and thermal rendering effects when interacting with virtual surfaces. Xia et al. [42] proposed a WTED incorporating a phase-change composite material (PCCM) heat sink. The results demonstrated that integrating the WTED with a PCCM heat sink achieved a temperature reduction of 2.5 K and enabled skin cooling durations of up to 10 min.

The convective heat loss from the lateral surfaces of thermoelectric legs in TEDs is another important factor influencing device performance [43]. Bjørk et al. [44] investigated internal heat losses in TEDs and demonstrated that a smaller ratio of the leg surface area to the cross-sectional area results in reduced convective heat loss from the side surfaces. Pang et al. [45] accounted for both lateral convective heat losses of thermoelectric legs and electrode contact thermal resistance, and showed that an optimal thermoelectric leg height exists for maximizing energy conversion efficiency. To further improve the accuracy and physical completeness of theoretical models, the Thomson effect has been recognized as a non-negligible contributor to thermoelectric behavior. Numerous studies have shown that neglecting this effect often leads to noticeable discrepancies between theoretical predictions and experimental results [46,47]. Huang et al. [48] examined the influence of the Thomson effect on temperature distributions and demonstrated that it can enhance the cooling efficiency of WTEDs. Manikandan and Kaushik [49] incorporated the Thomson effect into a heat transfer model of a two-stage TED and found that it reduces both the maximum power output and the efficiency of the device. Du and Wen [50] considered the Thomson effect in the internal thermal analysis of TEDs and developed three different Seebeck coefficient models to investigate the impact of Thomson heat on temperature prediction. Their results revealed that the temperature difference across a TED increases with increasing electrical load but decreases with increasing thermal load. Zhang et al. [51] developed a theoretical model for a micro-TED that incorporates both interfacial effects and the Thomson effect, and investigated the influences of current, filling factor, and thermoelectric leg height on cooling performance. The results showed that the Thomson effect significantly enhances the cooling performance of Bi_2_Te_3_-based micro-TEDs.

On the other hand, accurate modeling of heat conduction in skin tissue is essential for understanding human–device thermal interactions, elucidating skin thermal response mechanisms, and optimizing the design and reliability of related thermal functionalities. The Pennes bioheat equation [52] represents one of the most influential contributions to the study of heat transfer in biological tissues. Derived from the classical Fourier heat conduction law, it provides a simple yet effective framework and has been widely used. Subsequently, Chen and Holmes [53] investigated vascular structures, refined the bioheat formulation, and experimentally validated the applicability of the Pennes model, further promoting its extensive adoption. Although the Pennes bioheat equation has been widely applied, its formulation based on Fourier’s law assumes instantaneous heat propagation, which may limit its accuracy in describing transient thermal responses in skin tissue [54]. To overcome this limitation, Tzou [55] proposed the dual-phase lag (DPL) bioheat conduction model, which offers improved predictive capability for thermal responses in biological tissues. In the DPL framework, either the temperature gradient may precede the heat flux or the heat flux may precede the temperature gradient, thereby accounting for phase lag effects [56]. Ma et al. [57] compared the time-dependent thermomechanical responses of human skin predicted by the Pennes and DPL models, examined the influences of heat flux density and temperature gradient on blood perfusion, metabolism, and relaxation times, and demonstrated that the temperature equilibration process described by the DPL model is more effective than that of the Pennes model. Consequently, the DPL model is better suited for addressing more complex scenarios, such as thermal responses in multilayer biological tissues. Namakshenas and Mojra [58] applied the DPL model to investigate thermal damage and drug release in thermosensitive liposomes (TSLs), explicitly accounting for thermal inertia and microstructural interactions. Their theoretical predictions showed good qualitative and quantitative agreement with experimental observations, enabling more accurate temperature prediction and demonstrating superior performance compared with the classical Pennes bioheat model. In addition, Zhu et al. [59] developed a theoretical model for WTEDs that incorporates skin effects, analyzed the influences of skin on matched load resistance, optimal thermoelectric leg height, and filling factor, and derived a high-accuracy linearized solution suitable for power generation applications. They further established the optimal relationships among filling factor, thermoelectric leg height, and load resistance corresponding to maximum power output.

To ensure that WTEDs can fully and stably perform in human body temperature regulation, heat dissipation at the hot side represents another critical bottleneck. In wearable applications, stringent constraints on size, weight, and wearing comfort make the development of efficient, lightweight, and human-compatible heat dissipation strategies essential for long-term stable operation and effective thermal regulation [60,61]. Currently, heat dissipation approaches for WTEDs mainly include metal thin-film spreaders [62], metal fin heat sinks [63], and phase change material (PCM) cooling [64]. However, these conventional solutions often suffer from high rigidity, excessive weight, or limited cooling duration, making it difficult to simultaneously achieve efficient heat dissipation and wearing comfort. Hydrogels provide a promising alternative owing to their ability to enable efficient evaporative cooling through water storage, while offering low weight, mechanical softness, and good wearing comfort. Moreover, hydrogels can be regenerated by rehydration and reused cyclically [65,66,67], making them attractive for WTED heat dissipation. Park et al. [68] developed a heat sink composed of a solid silicone–hydrogel composite layer attached to the hot side of a thermoelectric device, achieving a modest cooling effect of approximately 3.8 K. Pu et al. [69] developed a temperature-sensitive polyhydrogel that mimics biological sweating to achieve high heat flux thermal management. The heat transfer coefficient of water vapor generated by the hydrogel is more than twice that of natural convection and radiation, allowing efficient operation even in environments with a relative humidity of 1% and significantly reducing the surface temperature of wearable electronic devices. Despite these advantages, the inherently low thermal conductivity of pure hydrogels limits rapid heat spreading from the hot side, thereby restricting evaporative cooling efficiency. To overcome this limitation, Wang et al. [70] employed a hydrogel–copper foam composite heat sink to enhance heat dissipation and increase the power output of a flexible thermoelectric generator, achieving an energy density of 95.4 μW/cm^2^ under natural convection—approximately 29 times higher than that obtained with a conventional planar heat sink. Zhang et al. [71] designed a bilayer heat sink composed of hydrogel and nickel foam, enabling a flexible thermoelectric cooler to maintain a temperature reduction of about 10 K for extended durations at an input current of 0.3 A, in both flat and bent configurations.

To investigate the dynamic thermal interactions between human skin and WTEDs in thermal tactile feedback, a dynamic thermal conduction framework is established by coupling DPL bioheat conduction in skin tissue with a WTED and a nickel foam-reinforced hydrogel heat sink. The framework enables accurate prediction of transient skin–device thermal responses and incorporates key mechanisms, including blood perfusion, metabolic heat generation, the Thomson effect, and side-surface convective heat losses of thermoelectric legs. A corresponding WTED prototype was fabricated and validated through human skin temperature regulation experiments, showing excellent agreement with theoretical predictions. On this basis, the device configuration was optimized, and controlled thermal tactile responses were demonstrated to replicate the thermal characteristics of common everyday objects. The remainder of this paper is organized as follows. Section 2 presents the theoretical model, Section 3 discusses the numerical and experimental results as well as applications in thermal tactile feedback, and Section 4 concludes the paper.

## 2. Dynamic Thermal Conduction Model for WTEDs Featuring Nickel Foam-Reinforced Hydrogel Heat Sink

### 2.1. Model Formulation

**Figure 1:**
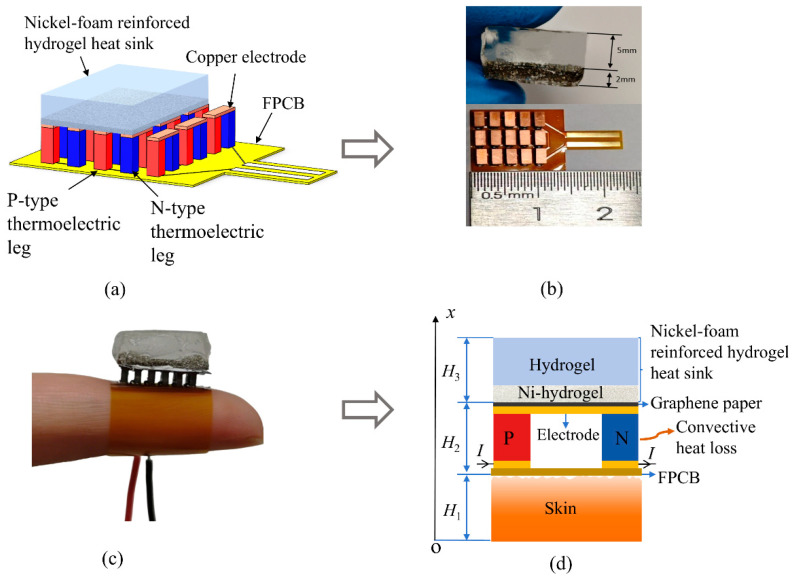
Schematic and experimental overview of the integrated skin–WTED system: (**a**) 3D structural schematic of the WTED; (**b**) photograph of the WTED and nickel foam-reinforced hydrogel heat sink; (**c**) experimental photograph of the WTED applied to the human skin; (**d**) thermal conduction model of the skin–WTED system.

Figure 1 presents photographs of a WTED integrated with human skin, along with a schematic of the skin–WTED heat conduction model. As shown in Figure 1a, the WTED consists of a flexible printed circuit board (FPCB), P- and N-type thermoelectric legs, and copper electrodes, with the legs electrically connected in series via the electrodes. Figure 1b shows a photograph of the fabricated WTED in this study (the fabrication procedure is described in Section 3), and the device has overall dimensions of 14 mm × 14 mm × 2.9 mm. Figure 1c presents an experimental photograph of the WTED equipped with a nickel foam-reinforced hydrogel heat sink, which is attached to the fingertip using polyimide (PI) tape. To investigate the influence of skin tissue and its physiological characteristics on the thermal behavior of the skin–WTED system, the model incorporates the effects of heat exchange induced by blood perfusion and metabolic heat generation. A multilayer thermal conduction model is established, consisting of the skin tissue, the WTED, and the hydrogel heat sink, as schematically illustrated in Figure 1d. A Cartesian coordinate system is defined with its origin located at the lower surface of the skin tissue. The thin interfacial layers, including copper electrodes and FPCB between the skin, thermoelectric legs, and hydrogel heat sink, are modeled as zero-thickness layers with finite thermal resistance to simplify the theory. The thicknesses of the skin tissue, thermoelectric legs, and hydrogel heat sink are denoted by *H*_1_, *H*_2_, and *H*_3_, respectively, and their corresponding temperature distributions are expressed as *T*_1_(*x*, *t*), *T*_2_(*x*, *t*), and *T*_3_(*x*, *t*).

Since the temperature difference between the hot and cold sides of the thermoelectric legs in WTEDs used for body temperature regulation is typically only on the order of several tens of Kelvin, the variations in thermoelectric material properties induced by temperature changes have a negligible effect on the cooling/heating performance of the WTED. Accordingly, the temperature dependence of thermoelectric material parameters is neglected in this study. Nevertheless, the model explicitly accounts for the Thomson effect as well as convective heat exchange between the thermoelectric legs and the surrounding environment. In addition, since the primary objective of this work is to predict the transient temperature response at the skin surface, the skin tissue is idealized as a homogeneous layer. Although multilayer skin models can provide more detailed temperature distributions within individual layers, their influence on the predicted surface temperature is generally limited, while substantially increasing the complexity of the theoretical formulation. As shown in Figure 1, the P-type and N-type thermoelectric legs share identical geometric configurations and are assumed to have symmetric material properties. The overall dimensions of the thermoelectric leg are 1 mm × 1 mm × 2.4 mm; therefore, heat conduction analysis is performed only for the P-type leg, from which the temperature distribution of the N-type leg can be readily obtained. The dimensions of the copper electrode are 3.5 mm × 1.2 mm. The hydrogel heat sink consists of a nickel foam–hydrogel composite substrate layer and an overlying pure hydrogel layer. To simplify the theoretical formulation, it is represented as an equivalent homogeneous medium with effective thermophysical properties determined using the equivalent thermal resistance method. Since the heat sink is separated from the skin by the WTED structure, its detailed internal temperature distribution has only a limited influence on the skin surface temperature response. This approximation preserves the dominant heat dissipation characteristics of the composite structure while significantly reducing model complexity. Accordingly, the effective thermal properties can be determined from the volume fractions and thermophysical properties of the constituent materials.

To capture the transient heat conduction in skin tissue, including thermal wave propagation and microstructural coupling effects, this study employs Tzou’s non-Fourier DPL model [55]. Combined with the classic Pennes bioheat equation [52], the governing DPL heat conduction equation for skin tissue is formulated as follows [72]:

(1) $$\left(1+\tau_{q}\frac{\partial }{\partial t}\right)\left\{\rho_{1}c_{1}\frac{\partial T_{1}\left(x,t\right)}{\partial t}+\eta_{b}\left[T_{1}\left(x,t\right)-T_{S}\right]-q_{m}\right\}=\lambda_{1}\left(1+\tau_{T}\frac{\partial }{\partial t}\right)\frac{\partial^{2}T_{1}}{\partial x^{2}}\;$$

where $\lambda_{1}$, $\rho_{1}$, and $c_{1}$ denote the thermal conductivity, density and specific heat capacity of the skin tissue, respectively. $\eta_{b}=\rho_{b}c_{b}w_{b}$, where $w_{b}$, $\rho_{b}$, and $c_{b}$ denote the blood perfusion rate, density, and specific heat, respectively. $\tau_{q}$ and $\tau_{T}$ are the phase lag parameters corresponding to the heat flux and the temperature gradient, respectively. $T_{S}$ and $q_{m}$ are the core body temperature and metabolic heat, while *x* and *t* represent the spatial coordinate and time.

Considering the Thomson effect in the thermoelectric legs of the WTED and the convective heat exchange between the thermoelectric legs and the surrounding environment, the one-dimensional transient heat conduction governing equation along the thickness direction of the thermoelectric legs can be expressed as [51,73]:

(2) $$\frac{\rho_{2}c_{2}}{\lambda_{2}}\frac{\partial T_{2}}{\partial t}=\frac{\partial^{2}T_{2}}{\partial x^{2}}-\frac{\tau I}{K_{2}H_{2}}\frac{\partial T_{2}}{\partial x}-\frac{hP}{K_{2}H_{2}}\left(T_{2}-T_{a}\right)+\frac{I^{2}R_{2}}{K_{2}H_{2}^{2}}$$

where $\rho_{2}$ and $c_{2}$ denote the density and specific heat capacity of the thermoelectric leg, respectively. *τ* denotes the Thomson coefficient, *I* is the electric current, *h* represents the heat convective coefficient at the side surfaces of the thermoelectric legs, *P* is the perimeter of the thermoelectric leg, and $T_{a}$ is the ambient temperature. $K_{2}=\lambda_{2}A_{2}/H_{2}$ denotes the thermal conductance of the thermoelectric leg, where $\lambda_{2}$ and $A_{2}$ are the thermal conductivity and cross-sectional area of the thermoelectric legs, respectively. $R_{2}=H_{2}/\left({\sigma_{2}A}_{2}\right)$ is the electrical resistance, with $\sigma_{2}$ denoting the electrical conductivity. The subscript “2” refers to parameters associated with the thermoelectric legs.

The heat transfer in the hydrogel heat sink can be described by the classical Fourier heat conduction equation [74]:

(3) $$\frac{\rho_{3}c_{3}}{\lambda_{3}}\frac{\partial T_{3}}{\partial t}=\frac{\partial^{2}T_{3}}{\partial x^{2}}$$

where $\lambda_{3}$, $\rho_{3}$, and $c_{3}$ denote the effective thermal conductivity, density, and specific heat capacity, respectively, and the subscript “3” indicates hydrogel-related parameters. The effective thermal conductivity $\lambda_{3}$ of the hydrogel heat sink is determined using the equivalent thermal resistance method, expressed as

(4a) $$\lambda_{3}=\frac{H_{3}}{r_{3}A}$$

where *A* is the cross-sectional area and $r_{3}$ is its total thermal resistance, given by

(4b) $$r_{3}=r_{Hy}+r_{Ni-hy}$$

Here, $r_{Hy}$ and $r_{Ni-hy}$ represent the thermal resistances of the upper pure hydrogel layer and the nickel foam–hydrogel composite substrate layer, respectively, which are defined as

(4c) $$r_{Ni-hy}=H_{Ni-hy}/\left(\lambda_{Ni-hy}A\right)$$

(4d) $$r_{Hy}=H_{Hy}/\left(\lambda_{Hy}A\right)$$

where $H_{Ni-hy}$ and $H_{Hy}$ denote the thicknesses of the composite substrate layer and the pure hydrogel layer, respectively. The effective thermal conductivity, $\lambda_{Ni-hy}$, can be evaluated as:

(4e) $$\lambda_{Ni-hy}=(1-\theta {)\lambda }_{Ni}+\theta \lambda_{Hy}$$

where θ is the porosity of the nickel foam, and $\lambda_{Ni}$ and $\lambda_{Hy}$ are the thermal conductivities of the nickel and hydrogel, respectively. Since the hydrogel is primarily composed of water and polyacrylamide, its thermal conductivity $\lambda_{Hy}$ can be estimated based on the volume fraction of water $\phi_{w}$ as:

(4f) $$\lambda_{Hy}=\phi_{w}\lambda_{w}+\left(1-\phi_{w}\right)\lambda_{p}$$

where $\lambda_{w}$ and $\lambda_{p}$ denote the thermal conductivities of water and polyacrylamide.

Similarly, the effective density $\;\rho_{3}$ and specific heat capacity $c_{3}$ of the nickel foam-reinforced hydrogel heat sink are expressed as

(4g) $$\rho_{3}=\frac{\rho_{Hy}V_{Hy}+\rho_{Ni}V_{Ni}}{V_{total}}$$

(4h) $$c_{3}=\frac{m_{Hy}c_{Hy}+m_{Ni}c_{Ni}}{m_{total}}$$

where $\rho_{Ni}$ and $\rho_{Hy}$ are the densities of nickel and hydrogel, respectively, and $c_{Ni}$ and $c_{Hy}$ are their corresponding specific heat capacities. Here, $V_{total}=V_{Ni}+V_{Hy}$ and $m_{total}=m_{Ni}+m_{Hy}$ denote the total volume and total mass of the hydrogel heat sink. $V_{Ni}$ and $V_{Ni-hy}$ denote the solid skeleton volume of nickel foam and the volume of the composite substrate layer, respectively. The total hydrogel volume $V_{Hy}$ can be further expressed as

(4i) $$V_{Hy}=AH_{Hy}+V_{Ni-hy}\theta$$

The initial conditions considered in this study assume that the skin tissue is initially at the human core body temperature, whereas the initial temperatures of the WTED and hydrogel heat sink are set to room temperature, i.e.,

(5a) $$T_{1}\left(x,0\right)=T_{S}$$

(5b) $$T_{2}\left(x,0\right)=T_{3}\left(x,0\right)=T_{a}$$

The temperature at the lower surface of the skin is maintained at the core body temperature, which can be expressed as

(6a) $$T_{1}\left(0,t\right)=T_{S}$$

Convective heat dissipation occurs between the upper surface of the hydrogel heat sink and the ambient environment, and the corresponding boundary condition is given by

(6b) $$Q_{3}\left(H,t\right)=K_{f}\left[T_{3}\left(H,t\right)-T_{a}\right]$$

where Q is the heat flux, $K_{f}=h_{f}A$, $h_{f}$ denotes the heat convective coefficient between the hydrogel heat sink and the surrounding environment, and $H=H_{1}+H_{2}+H_{3}$.

The continuity conditions of temperature and heat flux at the interfaces between the layers in the skin–WTED system can be expressed as:

(6c) $$T_{1}\left(H_{1},t\right)-T_{2}\left(H_{1},t\right)=\frac{Q_{2}\left(H_{1},t\right)}{K_{1ct}}$$

(6d) $$T_{2}\left(H_{12},t\right)-T_{3}\left(H_{12},t\right)=\frac{Q_{2}\left(H_{12},t\right)}{K_{2ct}}$$

(6e) $$Q_{1}\left(H_{1},t\right)=Q_{2}\left(H_{1},t\right)$$

(6f) $$Q_{2}\left(H_{12},t\right)=Q_{3}\left(H_{12},t\right)$$

Here, $H_{12}=H_{1}+H_{2}$, $K_{1ct}$ and $K_{2ct}$ represent the contact thermal conductances at the interfaces between the skin and the thermoelectric legs, and between the thermoelectric legs and the hydrogel heat sink, respectively.

The microscopic geometry and roughness of the skin surface result in an actual contact area that is much smaller than the nominal contact area, while the micro-voids filled with air at the interface introduce significant thermal contact resistance. The thermal contact conductance between the skin and the FPCB, denoted as $K_{ct}$, can be calculated using the following expression [75]:

(7a) $$K_{ct}=1.25\lambda_{ct}A\frac{\Delta a_{ct}}{\varepsilon_{ct}}{\left(\frac{P_{ct}}{H_{ct}}\right)}^{0.95}$$

where $P_{ct}$ is the contact pressure, and $H_{ct}$ denotes the microhardness, and $\lambda_{ct}$, $\varepsilon_{ct}$, and $\Delta a_{ct}$ denote the mean thermal conductivity, surface roughness, and surface slope of the contact interface, respectively, which are expressed as follows:

(7b) $$\lambda_{ct}=\frac{2\lambda_{1}\lambda_{B}}{\lambda_{1}+\lambda_{B}}$$

(7c) $$\varepsilon_{ct}=\sqrt{{\varepsilon_{1}}^{2}+{\varepsilon_{B}}^{2}}$$

(7d) $$\Delta a_{ct}=\sqrt{{\Delta a_{1}}^{2}+{\Delta a_{B}}^{2}}$$

Here, *ε* and ∆*a* denote the surface roughness and the slope of surface asperities, respectively, and the subscript “B” denotes the material properties of the FPCB layer. The thermal conductivity of the FPCB, $K_{B}$, can be calculated as $K_{B}=\lambda_{B}A/H_{B}$. Since the contact thermal resistance between the skin and the WTED is in series with the FPCB’s thermal resistance, the effective contact thermal conductance $K_{1ct}$ is given by $K_{1ct}={{(K}_{ct}^{-1}+K_{B}^{-1})}^{-1}$.

After soldering the upper surface of the thermoelectric legs with a copper sheet and bonding it to the hydrogel heat sink via a thin graphene paper, their effects are incorporated into the interfacial thermal resistance. The contact thermal conductance at the interface between the thermoelectric legs and the hydrogel heat sink is given by:

(8) $$K_{2ct}={\left(K_{cu}^{-1}+K_{gra}^{-1}+K_{c1}^{-1}+K_{c2}^{-1}\right)}^{-1}$$

where $K_{cu}=\lambda_{cu}A_{cu}/H_{cu}$ is the thermal conductance of the copper sheet, where $\lambda_{cu}$, $A_{cu}$, and $H_{cu}$ are its thermal conductivity, area, and thickness, respectively; $K_{gra}=\lambda_{gra}A_{gra}/H_{gra}$ is the thermal conductance of the graphene paper, with $\lambda_{gra}$, $A_{gra}$, and $H_{gra}$ representing its thermal conductivity, area, and thickness. The interfacial thermal conductances between the copper sheet and graphene paper, and between the graphene paper and the hydrogel heat sink, are denoted as $K_{c1}$ and $K_{c2}$, respectively, and can be calculated as $K_{c1}={A_{cu}/r}_{c1}$ and $K_{c2}={A_{cu}/r}_{c2}$, where $r_{c1}$ and $r_{c2}$ are the corresponding contact thermal resistances.

### 2.2. Analytical Temperature Solutions of the Skin–WTED System

By invoking the principle of superposition, the solution to the heat conduction governing equations for the skin-WTED system can be expressed as follows:

(9) $$T_{j}\left(x,t\right)=\varphi_{j}\left(x,t\right)+T_{j0},j=1\sim 3$$

where $T_{10}=T_{S}$ and $T_{20}=T_{30}=T_{a}$, $\varphi_{j}\left(x,t\right)\;\left(j=1\sim 3\right)$ denotes the transient temperature solutions for the individual layers. Substituting Equation (9) into the governing equations for the skin, thermoelectric legs, and hydrogel heat sink, given by Equations (1)–(3), yields:

(10a) $$\left(1+\tau_{q}\frac{\partial }{\partial t}\right)\left[\rho_{1}c_{1}\frac{\partial \varphi_{1}\left(x,t\right)}{\partial t}+\eta_{b}\varphi_{1}\left(x,t\right)-q_{m}\right]=\lambda_{1}\left(1+\tau_{T}\frac{\partial }{\partial t}\right)\frac{\partial^{2}\varphi_{1}\left(x,t\right)}{\partial x^{2}}$$

(10b) $$\frac{1}{\beta_{2}}\frac{\partial \varphi_{2}\left(x,t\right)}{\partial t}=\frac{\partial^{2}\varphi_{2}\left(x,t\right)}{\partial x^{2}}-\frac{\tau I}{K_{2}H_{2}}\frac{\partial \varphi_{2}\left(x,t\right)}{\partial x}-\frac{hP}{K_{2}H_{2}}\varphi_{2}+\frac{I^{2}R_{2}}{K_{2}H_{2}^{2}}\;$$

(10c) $$\frac{1}{\beta_{3}}\frac{\partial \varphi_{3}\left(x,t\right)}{\partial t}=\frac{\partial^{2}\varphi_{3}\left(x,t\right)}{\partial x^{2}}$$

with $\beta_{j}$ = $\lambda_{j}$/($\rho_{j}c_{j}$).

By combining Equations (5) and (9), the initial conditions of the problem can be written as

(11) $$\varphi_{j}\left(x,0\right)=0,j=1\sim 3$$

Substituting Equation (9) into the initial, boundary and continuity conditions defined in Equation (6a–f) leads to:

(12a) $$\varphi_{1}\left(0,t\right)=0$$

(12b) $$Q_{1}\left(H_{1},t\right)=Q_{2}\left(H_{1},t\right)$$

(12c) $$\varphi_{1}\left(H_{1},t\right)-\varphi_{2}\left(H_{1},t\right)=\frac{Q_{1}\left(H_{1},t\right)}{K_{1ct}}-\left(T_{S}-T_{a}\right)$$

(12d) $$Q_{2}\left(H_{12},t\right)=Q_{3}\left(H_{12},t\right)$$

(12e) $$\varphi_{2}\left(H_{12},t\right)-\varphi_{3}\left(H_{12},t\right)=\frac{Q_{2}\left(H_{12},t\right)}{K_{2ct}}$$

(12f) $$Q_{3}\left(H,t\right)=K_{f}\varphi_{3}\left(H,t\right)$$

The transient temperature distribution $\varphi_{j}\left(x,t\right)$ can be obtained using the Laplace transform, defined as [76]

(13) $$L\left[\varphi_{j}\left(x,t\right)\right]={\bar{\varphi }}_{j}\left(x,s\right)=\int_{0}^{\infty }\varphi_{j}\left(x,t\right)e^{-st}dt$$

Applying the Laplace transform to the temperature Equation (10a–c) results in:

(14a) $$\frac{\partial^{2}{\bar{\varphi }}_{1}}{\partial x^{2}}-\frac{\rho_{1}c_{1}s+\eta_{b}}{\Omega }{\bar{\varphi }}_{1}+\frac{q_{m\;}}{\Omega s}=0$$

(14b) $$\frac{\partial^{2}{\bar{\varphi }}_{2}}{\partial x^{2}}-\frac{\tau I}{K_{2}H_{2}}\frac{\partial {\bar{\varphi }}_{2}}{\partial x}-\left(\frac{hP}{K_{2}H_{2}}+\frac{s}{\beta_{2}}\right){\bar{\varphi }}_{2}+\frac{I^{2}R_{2}}{K_{2}H_{2}^{2}s}=0$$

(14c) $$\frac{\partial^{2}{\bar{\varphi }}_{3}}{\partial x^{2}}-\frac{s}{\beta_{3}}{\bar{\varphi }}_{3}=0$$

where $\Omega =\frac{\lambda_{1}(1+\tau_{T}s)}{1+\tau_{q}s}$.

After applying the Laplace transform, Equation (12a–f) take the following form:

(15a) $${\bar{\varphi }}_{1}\left(0,s\right)=0$$

(15b) $${\bar{Q}}_{1}\left(H_{1},s\right)={\bar{Q}}_{2}\left(H_{1},s\right)$$

(15c) $${\bar{\varphi }}_{1}\left(H_{1},s\right)-{\bar{\varphi }}_{2}\left(H_{1},s\right)=\frac{{\bar{Q}}_{1}\left(H_{1},s\right)}{K_{1ct}}-\frac{\left(T_{S}-T_{a}\right)}{s}$$

(15d) $${\bar{Q}}_{2}\left(H_{12},s\right)={\bar{Q}}_{3}\left(H_{12},s\right)$$

(15e) $${\bar{\varphi }}_{2}\left(H_{12},s\right)-{\bar{\varphi }}_{3}\left(H_{12},s\right)=\frac{{\bar{Q}}_{2}\left(H_{12},s\right)}{K_{2ct}}$$

(15f) $${\bar{Q}}_{3}\left(H,s\right)=K_{f}{\bar{\varphi }}_{3}\left(H,s\right)$$

Equation (14a–c) have solutions of the following form:

(16a) $${\bar{\varphi }}_{1}=B_{11}e^{w_{1}x}+B_{12}e^{-w_{1}x}+\gamma_{1}q_{m\;}$$

(16b) $${\bar{\varphi }}_{2}=B_{21}e^{w_{21}x}+B_{22}e^{w_{22}x}+\gamma_{2}I^{2}R_{2}$$

(16c) $${\bar{\varphi }}_{3}=B_{31}e^{w_{3}x}+B_{32}e^{-w_{3}x}$$

where $B_{jk}\;\left(j=1\sim 3,\;k=1,\;2\right)$ are the undetermined coefficients, and

(17a) $$\;\gamma_{1}=\frac{1}{s\left(\eta_{b}+s\rho_{1}c_{1}\right)}$$

(17b) $$\gamma_{2}=\frac{\beta_{2}}{H_{2}s\left(hP\beta_{2}+K_{2}H_{2}s\right)}$$

(17c) $$w_{1}=\sqrt{\frac{\eta_{b}+s\rho_{1}c_{1}}{\Omega }}$$

(17d) $$w_{21},\;w_{22}=\frac{\tau I}{{2K}_{2}H_{2}}\pm \sqrt{{\left(\frac{\tau I}{{2K}_{2}H_{2}}\right)}^{2}+\left(\frac{hP}{K_{2}H_{2}}+\frac{s}{\beta_{2}}\right)}$$

(17e) $$w_{3}=\sqrt{\frac{s}{\beta_{3}}}$$

By substituting Equation (16a–c) into the boundary and continuity conditions defined in Equation (15a–f), a set of linear algebraic equations for determining the coefficients $B_{jk}$ is obtained:

(18a) $$B_{11}+B_{12}=-\gamma_{1}q_{m\;}$$

(18b) $$B_{11}{\Omega A_{1}w_{1}e}^{w_{1}H_{1}}-B_{12}{\Omega A_{1}w_{1}e}^{{-w}_{1}H_{1}}+B_{21}e^{w_{21}H_{1}}\left(\alpha I-2K_{2}H_{2}w_{21}\right)\;+B_{22}e^{w_{22}H_{1}}\left(\alpha I-2K_{2}H_{2}w_{22}\right)=-\alpha I\left(\gamma_{2}I^{2}R_{2}+\frac{T_{a}}{s}\right)$$

(18c) $$B_{11}e^{w_{1}H_{1}}\left(1+\frac{\Omega A_{1}w_{1}}{K_{1ct}}\right)+B_{12}e^{{-w}_{1}H_{1}}\left(1-\frac{\Omega A_{1}w_{1}}{K_{1ct}}\right)-B_{21}e^{w_{21}H_{1}}-B_{22}e^{w_{22}H_{1}}=\gamma_{2}I^{2}R_{2}-\gamma_{1}q_{m}-\frac{T_{S}-T_{a}}{s}$$

(18d) $$B_{21}e^{w_{21}H_{12}}\left(\alpha I-2K_{2}H_{2}w_{21}\right)+B_{22}e^{w_{22}H_{12}}\left(\alpha I-2K_{2}H_{2}w_{22}\right)+B_{31}e^{w_{3}H_{12}}K_{3}H_{3}w_{3\;}-B_{32}e^{{-w}_{3}H_{12}}K_{3}H_{3}w_{3}=-\alpha I\left(\gamma_{2}I^{2}R_{2}+\frac{T_{a}}{s}\right)$$

(18e) $$B_{21}e^{w_{21}H_{12}}\left(1-\frac{\alpha I-2K_{2}H_{2}w_{21}}{K_{2ct}}\right)+B_{22}e^{w_{22}H_{12}}\left(1-\frac{\alpha I-2K_{2}H_{2}w_{22}}{K_{2ct}}\right)-B_{31}e^{w_{3}H_{12}}-B_{32}e^{{-w}_{3}H_{12}}=-\gamma_{1}I^{2}R_{2}+\frac{\alpha I\left(\gamma_{2}I^{2}R_{2}+\frac{T_{a}}{s}\right)}{K_{2ct}}$$

(18f) $$B_{31}e^{w_{3}H}\left(-K_{3}H_{3}w_{3}-K_{f}\right)+B_{32}e^{{-w}_{3}H}\left(K_{3}H_{3}w_{3}-K_{f}\right)=0$$

Thus, the dynamic temperature distribution in the frequency domain, ${\bar{\varphi }}_{j}\left(x,s\right)$, can be determined. The corresponding temperature distribution in the time domain is then obtained by performing an inverse Laplace transform as [77]:

(19) $$L^{-1}\left[\bar{\varphi }\left(x,s\right)\right]=\varphi \left(x,t\right)=-\frac{e^{\delta t}}{t}\sum_{k=1}^{n}Im[\bar{\varphi }\left(x,s\right)]sin(\frac{n\pi }{2})$$

where $s=\delta +\frac{n\pi }{2t}i$ and δ=3.5/t.

## 3. Numerical Results and Discussions

This section first assesses the accuracy and validity of the proposed dynamical thermal conduction model for the WTED through a comparison between theoretical predictions and experimental measurements. Building on this validation, the effects of key structural and dimensional parameters of the WTED on the transient temperature response of the skin surface are systematically analyzed. Finally, by combining theoretical modeling with experimental results, the potential of the WTED for reproducing thermal stimuli associated with thermal tactile perception is discussed.

### 3.1. Experimental Validation of the Analytical Model

To validate the proposed theoretical model, a WTED integrated with a nickel foam-reinforced hydrogel heat sink was fabricated, and wearable experiments were carried out to evaluate its capability for regulating human skin temperature. The device employs a FPCB as the substrate, which serves as a compliant supporting layer for the thermoelectric module. The thermoelectric legs are fabricated from Bi_2_Te_3_ and soldered onto the substrate electrodes to establish electrical interconnections. The opposite ends of the thermoelectric legs are soldered to copper electrodes, which are subsequently bonded to the hydrogel heat sink, enabling efficient heat conduction and conformal attachment. An image of the fabricated WTED is presented in Figure 1a, and the relevant material properties and dimensional parameters are listed in Table 1.

The fabrication process of the hydrogel heat sink is outlined as follows. First, acrylamide (AM), agar powder, and N,N’-methylenebisacrylamide (MBA) are dissolved in deionized water and thoroughly stirred to obtain a homogeneous precursor solution. The solution is then placed in a temperature-controlled oven to undergo a hydrothermal reaction. Upon completion, the resulting solution is poured into a mold preloaded with nickel foam, allowing it to fully infiltrate the porous structure of the nickel foam scaffold. Subsequently, an ammonium persulfate solution is introduced to initiate polymerization, after which the mold is sealed and cured in the temperature-controlled oven. The fabricated hydrogel heat sink is shown in Figure 1b, and the corresponding material properties and dimensional parameters are provided in Table 2. Furthermore, the nickel foam is assumed to have a porosity of θ=95%, indicating that 95% of the volume consists of pores filled with hydrogel, while the remaining 5% is occupied by the nickel skeleton. The thermal conductivities of water and polyacrylamide are taken as $\lambda_{w}=$ 0.6 W/mK and $\lambda_{p}$= 0.21 W/mK, respectively. The volume fraction of water in the hydrogel is taken as $\phi_{w}\;$= 83%. The interfacial thermal contact resistances $r_{c1}$ and $r_{c2}$ are taken as $3.28\;\times \;10^{-5}$ m^2^ K/W and $2.01\;\times \;10^{-4}$ m^2^ K/W, respectively [78].

The fabricated WTED is bonded to the hydrogel heat sink using a graphene thermal adhesive pad, forming the WTED integrated with the hydrogel heat sink, as shown in Figure 1c. During prolonged operation, the hydrogel heat sink may experience moisture loss due to sustained heat absorption, leading to a reduction in its heat dissipation performance; in such cases, the hydrogel heat sink can be readily detached from the adhesive pad and replaced. In the numerical simulations of the theoretical model, the thermophysical properties and dimensional parameters of the skin tissue are also required, and their values are summarized in Table 3. The blood perfusion rate, density, and specific heat are taken as $w_{b}=0.005\;mL/mL/s$, $\rho_{b}=1060\;kg/m^{3}$, and $c_{b}=3770\;J/kg/K$ [72], respectively. Unless otherwise stated, all subsequent numerical calculations are performed using the parameters listed in Table 1, Table 2 and Table 3.

**Figure 2:**
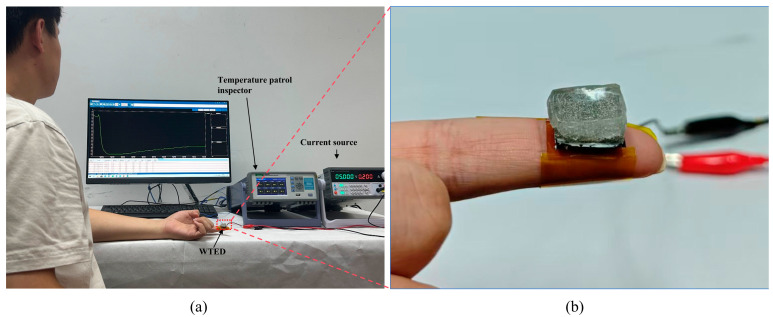
Wearable experiments of the WTED. (**a**) experimental setup for system characterization; (**b**) magnified view of the WTED.

As shown in Figure 2, the experimental setup for measuring skin temperature consists of a WTED integrated with a hydrogel heat sink, worn on the fingertip. The fabricated WTED was powered using a benchtop power supply (model SS-L305SPD, A-BF Electronics Co., Dongguan, China, accuracy 0.001 A/0.001 V). The temperature signals were acquired using a temperature monitoring instrument (model DC5508H, Zhongxiang Instrument Co., Zhongshan, China, accuracy ±0.2 K) and were simultaneously recorded and displayed on a computer via dedicated software, enabling real-time monitoring and analysis of the skin surface temperature dynamics.

All human skin experiments were conducted using a single healthy 27-year-old male participant (the first author of this study). The participant provided informed consent prior to the experiments. The ambient temperature was maintained at 293 K using an air-conditioning system. Prior to each test, the participant remained seated in the controlled environment for 15 min to allow the skin temperature to stabilize. The contact pressure between the WTED and the fingertip was maintained at approximately 1.6 kPa to ensure consistent thermal contact conditions. Each experiment was independently repeated five times (*n* = 5), and the results are presented as mean values with error bars. Sufficient rest intervals were provided between consecutive tests to allow the fingertip temperature to recover close to its initial condition before the subsequent measurement.

The skin surface temperature was measured using a spherical K-type thermocouple with a junction radius of 0.2 mm inserted between the skin and the WTED. Although the insertion of the thermocouple may introduce a small measurement uncertainty by slightly altering the local thermal contact conditions, this influence is expected to be limited because the thermocouple contact area is negligible compared with the overall skin–WTED contact area. In addition, the flexible PI-based substrate conforms well to the skin surface and possesses a relatively low thermal conductivity, which helps maintain uniform thermal contact and reduces thermocouple-induced disturbance. Previous studies have further shown that contact-pressure-induced temperature deviations in skin-temperature measurements are generally below 0.5 K [82]. Therefore, the measurement error associated with the thermocouple is expected to remain within the sub-degree range.

**Figure 3:**
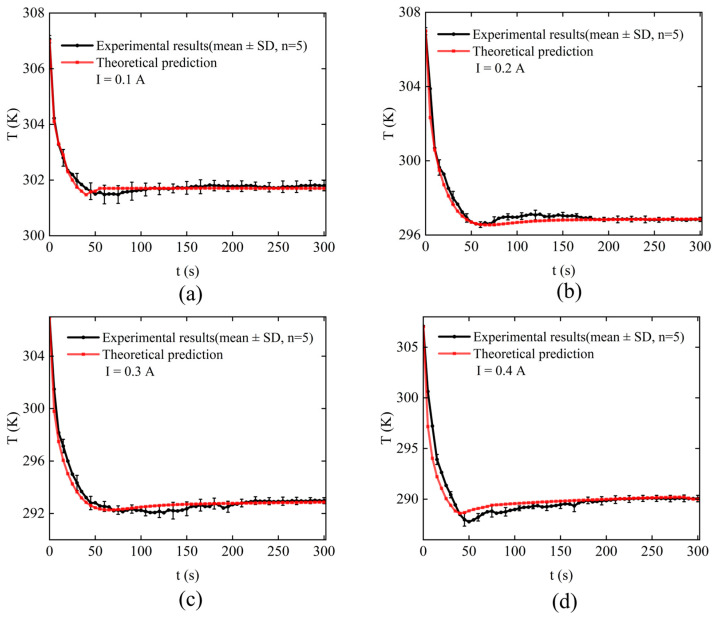
Comparison between theoretical predictions and experimental results of skin surface temperature under different cooling currents: (**a**) *I* = 0.1 A, (**b**) *I* = 0.2 A, (**c**) *I* = 0.3 A, (**d**) *I* = 0.4 A.

Figure 3 shows the temporal evolution of skin surface temperature under different cooling currents, comparing theoretical predictions with experimental measurements. Figure 3a–d correspond to cooling currents of 0.1, 0.2, 0.3, and 0.4 A, respectively. The results indicate that the theoretical model accurately captures the temperature trends observed experimentally: once the WTED is powered, the skin surface temperature drops immediately, reaching a minimum within approximately 30–60 s. A slight temperature rebound follows, which becomes more pronounced at higher currents. For example, at *I* = 0.4 A (Figure 3d), the magnitude of this rebound is significantly larger than that observed under lower currents.

Moreover, increasing the current enhances the WTED’s cooling performance, resulting in a more substantial reduction in skin surface temperature. Specifically, as the current increases from 0.1 A to 0.4 A, the theoretically predicted skin temperature at ~300 s (near steady state) decreases from 301.7 K to 290.0 K. Overall, the theoretical predictions show good agreement with the experimental data in terms of both transient evolution and steady-state temperature. The corresponding mean absolute error (MAE) values are 0.176, 0.227, 0.401, and 0.579 K for cooling currents of 0.1, 0.2, 0.3, and 0.4 A, respectively. All MAE values are below 0.6 K, demonstrating the capability of the proposed model to accurately predict the skin temperature response under different operating conditions. Therefore, the model provides a reliable framework for analyzing the thermal behavior of WTEDs and guiding subsequent device optimization.

### 3.2. Dynamic Thermal Response of the Skin–WTED System

This section presents a numerical analysis of the effects of cooling current, hydrogel heat sink, skin–WTED contact pressure, filling factor *F* (the cross-sectional area fraction of P- and N-type thermoelectric legs in the WTED), and thermoelectric leg height on the dynamic temperature response of the skin surface. The study primarily focuses on the cooling regulation of skin temperature by the WTED, motivated by two considerations: first, lowering the skin temperature to a desired level is more challenging than heating it, which can generally be achieved via Joule heating in flexible devices; second, this focus lays the groundwork for subsequent applications of WTED in thermal tactile perception, since objects in contact with the human body are typically much cooler than the skin surface.

**Figure 4:**
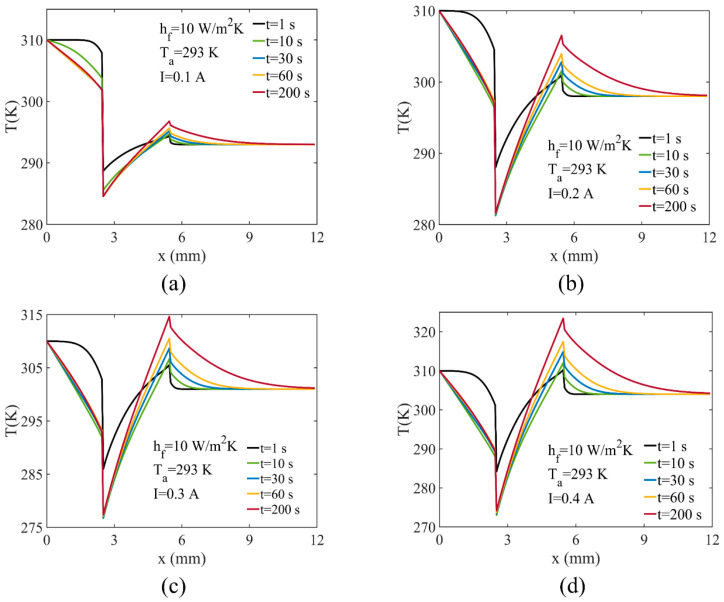
Dynamic temperature fields of the skin–WTED system under various cooling currents: (**a**) *I* = 0.1 A, (**b**) *I* = 0.2 A, (**c**) *I* = 0.3 A, and (**d**) *I* = 0.4 A.

Figure 4 shows the temporal evolution of temperature distributions within the human skin, the WTED, and the hydrogel heat sink under various cooling currents. At the initial stage of the current application (e.g., *t* = 1 s), the temperatures of the thermoelectric legs respond rapidly, forming pronounced gradients at both ends. At this time, only the regions of the skin and hydrogel heat sink adjacent to the WTED contact interface exhibit slight temperature changes. Due to the contact thermal resistances at both the skin–WTED and WTED–hydrogel heat sink interfaces, sharp temperature jumps occur at these locations. In particular, the larger contact resistance between the skin and WTED results in a more significant temperature discontinuity; under currents of *I* = 0.2–0.4 A at *t* = 1 s, the temperature difference can exceed 17 K. As time progresses, the temperature jump at the skin–WTED interface gradually diminishes, yet numerical results indicate that even at t = 60 s, this difference remains above 14 K. When the current increases from 0.1 A to 0.4 A, the minimum temperatures at the WTED cold end are 284.8 K, 281.6 K, 277.4 K, and 274.2 K, corresponding to skin surface temperatures of 301.8 K, 297.0 K, 292.9 K, and 289.5 K, respectively. Simultaneously, the initially nonlinear temperature distribution within the skin gradually evolves toward an approximately linear profile.

For the hydrogel heat sink, at low cooling currents such as *I* = 0.1 A, the overall temperature variation is minimal, and the convective surface in contact with the environment remains near the initial room temperature. As the cooling current increases, the hydrogel heat sink’s overall temperature rises, yet it continues to efficiently absorb the heat generated by the WTED, maintaining effective thermal management. Experimental results show that the hydrogel heat sink can sustain stable heat dissipation for approximately two hours of continuous operation; afterward, gradual water loss reduces its heat storage and dissipation performance, requiring replacement.

**Figure 5:**
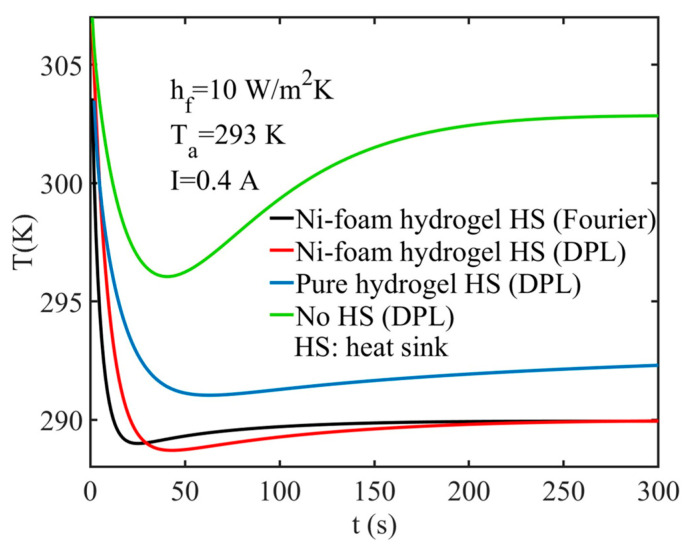
Comparison of predicted skin surface temperature between the DPL Pennes bioheat model and the classical Pennes bioheat model for different heat sinks.

Figure 5 compares the predicted skin surface temperature obtained using the DPL Pennes bioheat model and the Fourier law-based classical Pennes bioheat model for different heat sinks. The DPL model predicts a minimum transient temperature of 288.70 K at approximately 43 s, whereas the classical Pennes model reaches a minimum temperature of 288.99 K at approximately 25 s. Although the minimum temperatures predicted by the two models are nearly identical, the cooling rate predicted by the DPL model is noticeably lower than that predicted by the classical Pennes model. This difference can be attributed to the phase lag effects incorporated in the DPL heat conduction model. In the present study, the phase lag times of the heat flux and temperature gradient are 6.83 s and 17.04 s, respectively. These finite phase lags imply that the heat flux does not respond instantaneously to changes in the temperature gradient, resulting in a delayed propagation of thermal disturbances during the initial cooling stage. Consequently, the heat transfer process exhibits a pronounced thermal inertia effect, leading to a slower cooling response and a delayed occurrence of the minimum temperature. Despite these differences, the minimum temperatures predicted by the two models differ by less than 0.3 K, indicating that the phase lag effects primarily influence the short-term temperature evolution while having only a limited impact on the overall temperature level. Since thermal tactile perception is governed mainly by the transient skin-temperature response immediately after contact, accurate prediction of the early-stage cooling process is particularly important. Therefore, the DPL model is adopted in this study, as it can account for the finite-speed thermal response of biological tissues and provide a more realistic description of transient heat transfer in the skin–WTED system.

To further evaluate the thermal performance of the proposed heat sink, a comparison was conducted among three configurations: nickel foam-reinforced hydrogel heat sink, pure hydrogel heat sink, and the case without any heat sink are also provided in Figure 5. The results indicate that the incorporation of nickel foam significantly enhances the effective thermal conductivity and heat-spreading capability of the hydrogel-based structure. Compared with the pure hydrogel heat sink, the nickel foam-reinforced configuration results in a reduction of approximately 2.3 K in both the minimum skin temperature and the steady-state temperature, indicating improved heat removal efficiency. More importantly, when compared with the case without a heat sink, the presence of the nickel foam-reinforced hydrogel heat sink leads to a reduction of approximately 7.3 K in the transient skin temperature and up to 12.8 K in the steady-state regime. These results demonstrate that the nickel foam reinforcement substantially enhances heat diffusion within the hydrogel matrix, thereby improving thermal dissipation from the WTED and achieving a more pronounced and stable cooling effect on the skin surface.

It should be noted that prolonged operation may lead to gradual water loss from the hydrogel heat sink due to evaporation. Nevertheless, our previous study [83] demonstrated that the nickel foam–reinforced hydrogel heat sink exhibits excellent dehydration–rehydration reversibility. After repeated dehydration–rehydration cycles, the hydrogel recovered nearly its original mass, with a maximum deviation of only 1.11% after rehydration. Therefore, the thermal performance of the hydrogel heat sink can be effectively restored through water replenishment, indicating good long-term reusability and stability.

**Figure 6:**
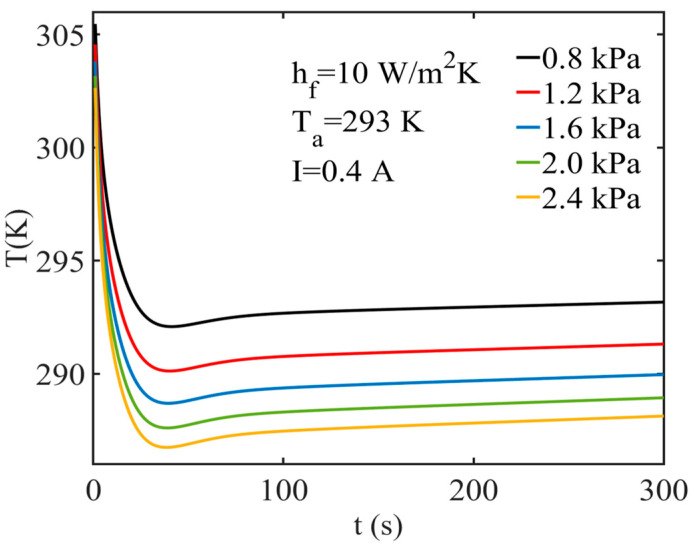
Effect of contact pressure between the skin and WTED on the skin surface temperature.

Another critical factor affecting the WTED’s skin temperature regulation is the contact pressure between the skin and the device. Higher contact pressure enhances conformity at the interface, reduces thermal resistance, and consequently decreases the temperature difference between the skin surface and the device. As shown in Figure 6, contact pressure has a pronounced effect on skin temperature. For instance, with a cooling current of 0.4 A, an ambient temperature of 293 K, and a heat convective coefficient of 10 W/m^2^ K, increasing the contact pressure from 0.8 kPa to 2.4 kPa lowers the skin surface temperature by approximately 5.1 K and accelerates the cooling rate. Balancing long-term wearing comfort with the need to minimize interfacial thermal resistance, a contact pressure of 1.6 kPa is selected as optimal for the skin–WTED interface in this paper.

**Figure 7:**
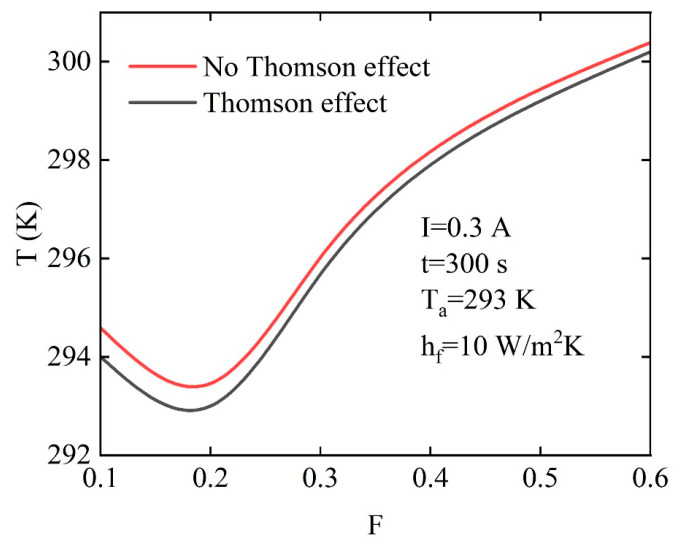
Effects of the Thomson effect and the filling factor of the WTED on the skin surface temperature.

Figure 7 presents the effects of the Thomson effect and filling factor of the WTED on the skin surface temperature under steady-state conditions (*t* = 300 s). The filling factor exerts a significant influence on the cooling performance of the WTED. With increasing filling factor, the skin surface temperature initially decreases and then increases, indicating the existence of an optimal value of *F* = 0.18 at which the minimum temperature is achieved. When the filling factor exceeds this optimum, the skin surface temperature rises rapidly. This trend can be attributed to an excessively high filling ratio, which hinders efficient heat conduction and dissipation from the hot side of the device to the hydrogel heat sink, leading to heat accumulation and a concomitant increase in the cold-side temperature of the WTED, thereby weakening its cooling capability. Furthermore, the numerical results reveal that for WTEDs based on Bi_2_Te_3_ thermoelectric materials, the Thomson effect does not affect the value of the optimal filling factor, but it provides a positive enhancement to the cooling performance. This enhancement is more pronounced at lower filling factors. For instance, at *F* = 0.2, inclusion of the Thomson effect results in an approximately 0.46 K lower skin surface temperature compared with the case in which it is neglected. These findings indicate that incorporating the Thomson effect into the model improves the predictive accuracy of WTED-based skin temperature regulation.

**Figure 8:**
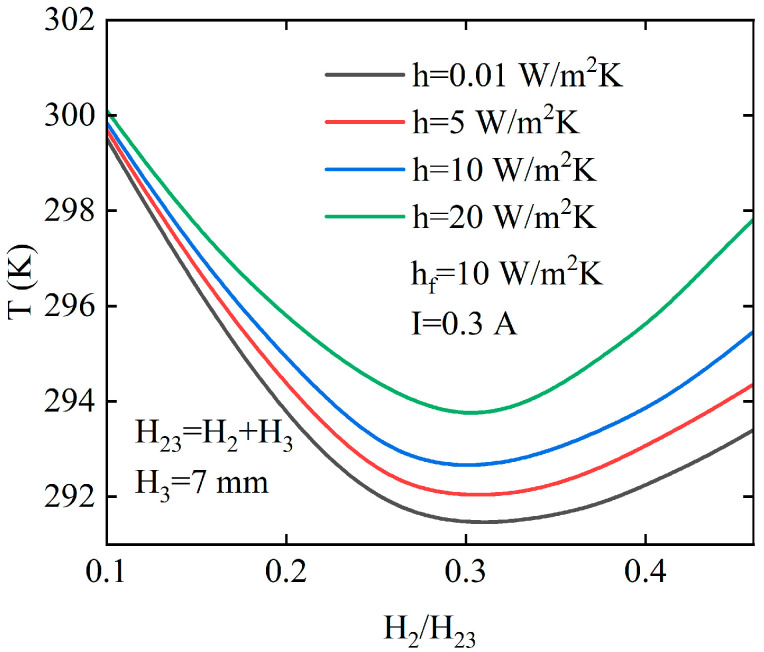
Effects of the thermoelectric leg height and side-surface heat convective coefficient of the WTED on the skin surface temperature.

Figure 8 depicts the influences of the thermoelectric leg height and convective heat loss from the leg side surfaces on the skin surface temperature. The results reveal that the cooling performance of the WTED exhibits a clear optimum with respect to the thermoelectric leg height. When the normalized leg height reaches $H_{2}/H_{23}=0.30$ (where $H_{23}=H_{2}+H_{3}$ denotes the total device height including the hydrogel heat sink, $h=10\;W/m^{2}K)$, the skin surface temperature attains its minimum. Under this condition, $H_{2}$ is approximately 3.0 mm, and the corresponding temperature is only 0.34 K lower than that obtained for $H_{2}$ = 2.4 mm. Considering the requirements of wearability and structural compactness, excessively large leg heights are undesirable; therefore, $H_{2}$ = 2.4 mm is selected as the thermoelectric leg height for device fabrication in this study. In addition, convective heat exchange between the thermoelectric legs and the surrounding environment has a pronounced effect on the cooling performance of the WTED. Taking $H_{2}$ = 2.4 mm as an example, when the side-surface heat convective coefficient h = 0.01, 5, 10, and 20 W/m^2^ K the corresponding skin surface temperatures are 291.76 K, 292.29 K, 292.90 K, and 294.21 K, respectively. Compared with the idealized case in which side-surface convective losses are neglected, the skin surface temperature increases by 2.45 K at h = 20 W/m^2^ K.

### 3.3. Application of the Model for Thermal Tactile Feedback

In this section, the developed skin–WTED heat conduction model is applied to study thermal tactile feedback. To assess the model’s applicability under different thermal conditions, three representative materials were selected: an iron block (high thermal conductivity solid), a polymethyl methacrylate (PMMA) plate (low thermal conductivity solid), and a common carbonated beverage (liquid) contained in an aluminum can or plastic bottle. These materials represent typical thermal properties and heat transfer mechanisms encountered in daily life, enabling analysis of the transient thermal response of the skin upon contact with different objects and evaluation of the model’s predictive accuracy. The experimental setup and the thermal tactile feedback system were consistent with the skin–WTED measurement system shown in Figure 2.

**Figure 9:**
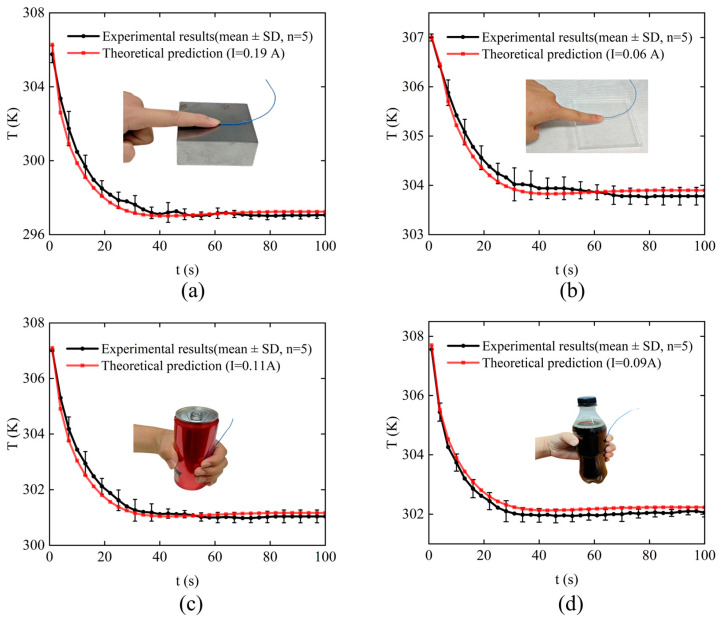
Comparison between theoretical predictions and experimental results for WTED-based thermal tactile feedback: (**a**) iron block, (**b**) PMMA plate, (**c**) carbonated beverage in an aluminum can, (**d**) carbonated beverage in a plastic bottle.

Figure 9 shows the temporal evolution of fingertip surface temperature when in contact with the three types of materials. Due to the pronounced differences in their thermal properties, the skin exhibits distinct heat transfer and temperature response behaviors. Both the initial rapid cooling phase and the subsequent steady-state phase differ in cooling rate and final temperature, which underlie the human ability to discriminate between the tactile sensations of different objects under the same ambient conditions [15]. The experimental results indicate that within the first ~10 s, the skin temperature drops rapidly from 307 K to approximately 300.5 K when contacting the highly conductive iron block, whereas it decreases only to 305.5 K when contacting the PMMA plate. The cooling rate for the liquid carbonated beverage lies between these two extremes. Since the volume of the tested object is much larger than that of the fingertip, its overall heat capacity is considerably higher than that of the skin. As a result, the object’s bulk temperature change during contact can be considered negligible, with significant temperature variations occurring only in the local contact region. Under these conditions, the steady-state temperature of the skin in contact with the object is primarily determined by the object’s thermal conductivity and the interfacial thermal resistance, with higher thermal conductivity leading to faster heat dissipation from the skin and a lower steady-state temperature. Specifically, the steady-state temperature reaches a minimum of ~297.2 K for the metal block and a maximum of 303.8 K for the PMMA plate.

Figure 9c and Figure 9d compare the thermal tactile responses of the skin when contacting the same liquid carbonated beverage packaged in an aluminum can and a plastic bottle, respectively. The results show that the packaging material noticeably influences the temporal evolution of fingertip temperature. Compared with the plastic bottle, contact with the aluminum can produces a faster initial decrease in skin surface temperature. In addition, the plastic-bottle case exhibits a temperature evolution characterized by a rapid drop followed by a slight recovery before reaching a steady state, whereas the aluminum-can case shows a rapid decrease followed by a monotonic approach to steady state. At steady state, the fingertip temperature in contact with the aluminum can is approximately 0.8 K lower than that observed for the plastic bottle. These differences are primarily attributed to variations in the effective interfacial thermal resistance between the fingertip and the beverage. Owing to its much lower thermal resistance, the aluminum can facilitates more efficient heat transfer from the skin to the liquid than the plastic bottle. This result indicates that even with identical internal liquids, subtle differences in packaging materials and interface thermal properties can give rise to thermal tactile variations that are potentially perceptible to humans. Such sensitivity underscores the need for high-fidelity modeling and reconstruction of thermal tactile sensations in virtual and augmented reality systems.

Figure 9 also presents the theoretical predictions of skin temperature responses underlying thermal tactile feedback generated by the WTED. In the simulations, the device driving current was set to 0.19 A, 0.06 A, 0.11 A, and 0.09 A, corresponding to the thermal responses observed when the skin contacted an iron block, a PMMA plate, an aluminum can, and a bottled carbonated beverage, respectively. For instance, a driving current of 0.19 A replicates the rapid cooling and steady-state temperature observed during contact with the iron block, while 0.06 A captures the slower temperature decrease observed during contact with the PMMA plate. The results show that the WTED-induced skin temperature profiles closely match the experimental measurements, accurately reproducing both the initial transient cooling and the steady-state temperatures for each object. The calculated MAE values between the theoretical predictions and experimental mean temperatures are 0.359, 0.167, 0.221, and 0.230 K for the iron block, PMMA plate, carbonated beverage in an aluminum can, and carbonated beverage in a plastic bottle, respectively. These relatively small errors further demonstrate the predictive capability of the proposed model over the entire transient response. Based on this validated framework, the WTED can reproduce skin temperature responses associated with contact with different objects by adjusting the cooling current, thereby providing stable and controllable thermal tactile feedback.

## 4. Conclusions

This paper presents a dynamic thermal conduction analytical model for WTEDs, which incorporates skin physiological characteristics and a nickel foam-reinforced hydrogel heat sink. The model incorporates key physical mechanisms, including skin blood perfusion and metabolic heat generation, the DPL heat conduction effect, the Thomson effect in the WTED, convective heat losses from the thermoelectric legs, and interfacial contact thermal resistance. A WTED prototype with the proposed hydrogel heat sink was fabricated and validated through human skin temperature regulation experiments, showing close agreement with theoretical predictions and thereby confirming the model’s accuracy and reliability. Based on the validated theoretical framework, key structural parameters of the WTED, including the filling factor and thermoelectric leg height, were optimized to enhance device performance. Using the minimum skin surface temperature as the optimization criterion, the optimal filling factor and normalized leg height were determined to be *F* = 0.18 and $H_{2}/H_{23}=0.30$, respectively. In addition, convective heat loss from the side surfaces of the thermoelectric legs raises the skin temperature. Compared with the ideal scenario without side-surface convection, the skin surface temperature increases by 2.45 K when the heat convective coefficient *h* = 20 W/(m^2^ K). Moreover, thermal tactile feedback was successfully demonstrated, enabling controlled reproduction of skin temperature responses associated with contact with common objects, including an iron block, a PMMA plate, and carbonated beverages in aluminum cans and plastic bottles. Overall, the proposed model provides a predictive foundation for the design and optimization of WTED-based thermal tactile systems, supporting precise skin temperature control and the future development of high-fidelity thermal perception.

## Figures and Tables

**Table 1:** Thermophysical properties and dimensional parameters of the WTED [38,59].

Description	FPCB	${Bi}_{2}{Te}_{3}$	Cu
Height (mm)	0.13	2.4	0.2
Density (kg/m^3^)	2255	7700	-
Specific heat (J/kg/K)	2684	200	-
Thermal conductivity (W/mK)	4	1.6	400
Seebeck coefficient (μV/K)	-	200	-
Thomson coefficient (μV/K)	-	80	-
Electrical conductivity (10^5^ S/m)	-	1.1	-
Heat convective coefficient (W/m^2^ K)	-	10	-

**Table 2:** Thermophysical properties and dimensional parameters of the nickel foam-reinforced hydrogel heat sink [75,79,80].

Description	Nickel Foam	Hydrogel	Hydrogel Heat Sink
Height (mm)	2	5	7
Density (kg/m^3^)	350	1070	1100
Specific heat (J/kg/K)	440	3768	3752
Thermal conductivity (W/mK)	90	0.535	0.866

**Table 3:** Thermophysical properties and dimensional parameters of skin [37,59,72,81].

Description	Values
Thickness(mm)	2.5
Density (kg/$m^{3}$)	971
Specific heat (J/kg/K)	2700
Metabolic heat generation rate (W/$m^{3}$)	368.1
Thermal conductivity (W/mK)	0.244
Phase lag parameter of heat flux (s)	6.83
Phase lag parameter of temperature gradient (s)	17.04
Surface roughness (μm)	21.69
Surface asperity slope (rad)	0.3
Microhardness (MPa)	0.1225
Contact pressure (kPa)	1.6

## Data Availability

The data supporting the findings of this study are available within the article and from the corresponding author upon reasonable request.

## Abbreviations

The following abbreviations are used in this manuscript:

Abbreviation	
AM	Acrylamide
AR	Augmented reality
DPL	Dual-phase lag
FPCB	Flexible printed circuit board
MAE	Mean absolute error
MBA	Methylenebisacrylamide
PCM	Phase change material
PCCM	Phase-change composite material
PMMA	Polymethyl methacrylate
TED	Thermoelectric device
TSLs	Thermosensitive liposomes
VR	Virtual reality
Subscripts	
a	Ambient environment
B	Flexible printed circuit board
b	Blood
c1	Contact interface between the electrode and graphene paper
c2	Contact interface between the graphene paper and hydrogel heat sink
ct	Contact thermal
f	Environmental convection
gra	Graphene paper
Hy	Hydrogel
m	Metabolic heat
Ni	Nickel foam
Ni-hy	Nickel foam–hydrogel composite substrate layer
p	Polyacrylamide
S	Core body temperature
w	Water
Symbol	Description
*A*	Cross-sectional area (mm^2^)
*c*	Specific heat (J/kg/K)
*H*	Height (mm) or microhardness (MPa)
*h*	Convection coefficient (W/m^2^ K)
*I*	Current (A)
*K*	Thermal conductance (W/K)
*m*	Mass (g)
*P*	Contact pressure (kPa) or perimeter (mm)
*Q*	Heat flux (W)
*q*	Metabolic heat (W/m^3^)
*R*	Electric resistance (Ω)
*r*	thermal resistance (K/W)
*T*	Temperature (K)
*t*	Time (s)
*V*	Volume (m^3^)
Greek letters	
α	Seebeck coefficient (V/K)
Δa	Asperity slope (rad)
ε	Surface roughness (μm)
λ	Heat conductivity (W/mK)
ρ	Density (kg/m^3^)
σ	Electric conductivity (S/m)
τ	Thomson coefficient (V/K) or phase lag coefficients (s)
φ	Transient temperature solutions (K)
ϕ	Volume fraction (%)
ω	Perfusion rate of biological tissue (mL/mL/s)
θ	Porosity (%)
